# Crystal structure of {2,2′-[*N*,*N*′-bis­(pyridin-2-yl­meth­yl)cyclo­hexane-*trans*-1,2-diyldi(nitrilo)]di­acetato}­cobalt(III) hexa­fluorido­phosphate

**DOI:** 10.1107/S2056989015005149

**Published:** 2015-03-21

**Authors:** Craig C. McLauchlan, Daniel S. Kissel, Albert W. Herlinger

**Affiliations:** a Department of Chemistry, Illinois State University, Campus Box 4160, Normal, IL 61790-4160, USA; b Department of Chemistry and Biochemistry, Loyola University Chicago, Chicago, IL 60626, USA

**Keywords:** crystal structure, polyamino­carb­oxy­lic acid, cobalt(III), chelating ligand

## Abstract

The title compound [Co(C_22_H_26_N_4_O_4_)]PF_6_, commonly known as [Co(bpcd)]PF_6_, where bpcd^2−^ is derived from the historical ligand name *N*,*N*′-bis­(2-pyridyl­meth­yl)-*trans*-1,2-di­amino­cyclo­hexane-*N*,*N*′-di­acetate, crystallized by slow evaporation of a saturated aceto­nitrile solution in air. The cation of the hexa­fluorido­phosphate salt has the Co^III^ atom in a distorted octa­hedral coordination geometry provided by an N_4_O_2_ donor atom set. The acetate groups, which are oriented trans with respect to each other, exhibit monodentate coordination whereas the pyridyl N atoms are coordinating in a cis configuration. The geometry of the cation is compared to the geometries of other di­amino di­acetate complexes with Co^III^.

## Abstract 2

In the title compound, a Co^III^ center is coordinated by four N atoms and two O atoms, with the monodentate acetate groups of the ligand oriented trans with respect to each other, whereas the pyridine N atoms are coordinated in a cis configuration.

## Chemical context   

Polyamino­carb­oxy­lic acids are of considerable inter­est as complexation reagents for a variety of metal ions in a wide range of applications (Weaver & Kappelmann, 1964 ▸; Weiner & Thakur, 1995 ▸; Caravan *et al.*, 1997*a* ▸,*b* ▸; Geraldes, 1999 ▸; Heitzmann *et al.*, 2009 ▸). The title compound, [Co(bpcd)]PF_6_, (I), was prepared from *N*,*N*′-bis­(2-pyridyl­meth­yl)-*trans*-1,2-di­amino­cyclo­hexane-*N*,*N*′-di­acetic acid (H_2_bpcd), a sym­metrically disubstituted polyamino­carb­oxy­lic acid featuring a chiral *trans*-di­amino­cyclo­hexane backbone.
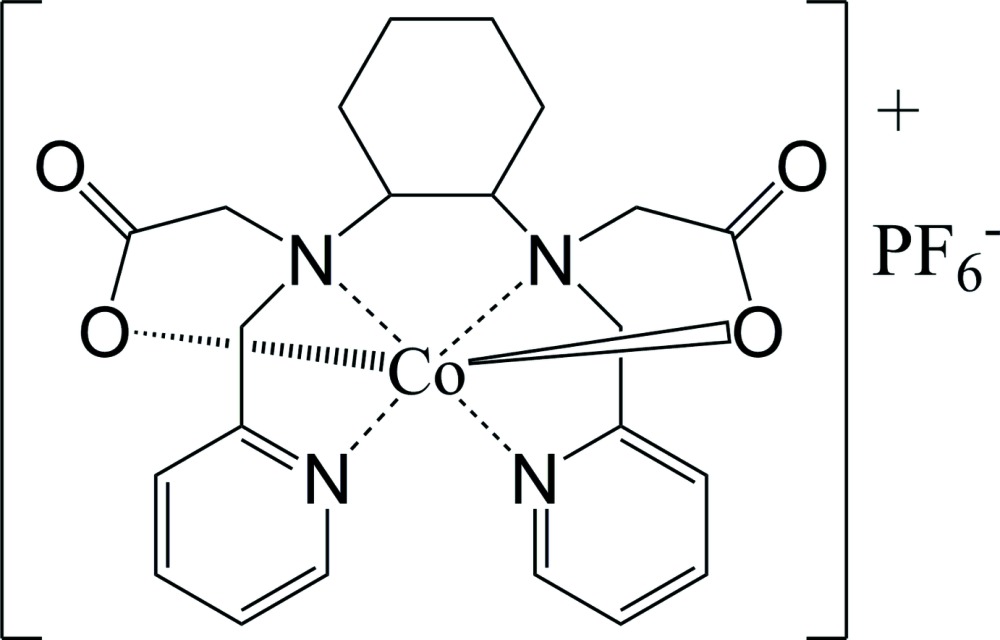


**Figure 1:**
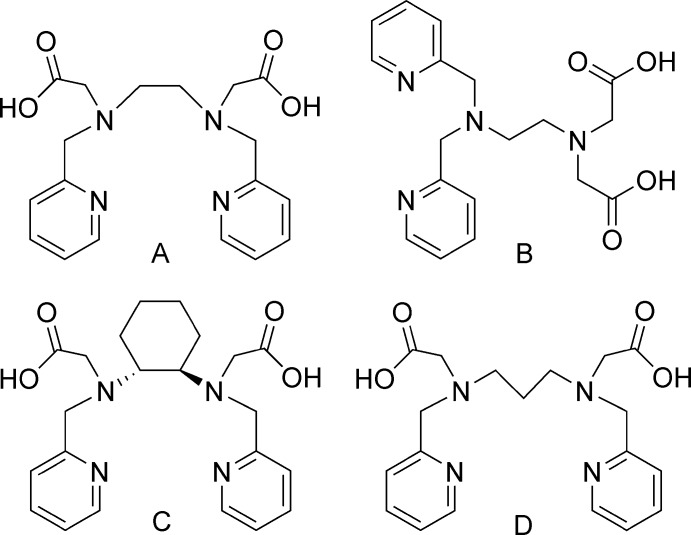
The di­amino di­acetic acids, H_2_bped (A) and *gem*-H_2_bped (B), where bped stands for bis­(2-pyridyl­meth­yl)-1,2-di­amino­ethane di­acetate, H_2_bpcd (C), and H_2_bppd (D), where bppd stands for bis­(2-pyridyl­meth­yl)-1,3-di­amino­propane di­acetate.

The ligand precursor, H_2_bpcd, belongs to a relatively small group of di­amino di­acetic acids that contain softer aromatic nitro­gen donor groups (Fig. 1 ▸) (Caravan *et al.*, 1997*a* ▸; Heitzmann *et al.*, 2009 ▸; Kissel *et al.*, 2014 ▸). The preorganized ligand precursor H_2_bpcd is of inter­est as a novel candidate for selective and efficient actinide(III)/lanthanide(III) separations. Preorganization of a ligand can reduce the pre-orientation energy required for metal ion complexation and provide improved metal–ligand complex stability (Rizkalla *et al.*, 1987 ▸; Choppin *et al.*, 2006 ▸; Ogden *et al.*, 2012 ▸). The addition of aromatic functionalities, such as pyridine and pyrazine, may increase ligand selectivity for softer metal ions and provide greater stability towards radiolysis (Heitzmann *et al.*, 2009 ▸). The members of this group of di­acetic acids, however, differ in the nature of the di­amine backbone.

The ethyl­enedi­amine backbone is a classic scaffold that has been used for the construction of many polydentate ligands. The amine N atoms are ideal for functionalization, which allows different donor atom groups to be incorporated into a ligand’s design. The close proximity of the di­amine nitro­gens also maximizes the number of possible five- and six-membered chelate rings capable of forming upon metal ion complexation. H_2_bped (A) is a hexa­dentate 2-pyridyl­methyl-substituted di­acetic acid based on this classic scaffold (Lacoste *et al.*, 1965 ▸; Caravan *et al.*, 1997*a* ▸). *gem*-H_2_bped (B) is a very closely related 2-pyridyl­methyl-substituted di­acetic acid that is also based on the ethyl­enedi­amine scaffold. In this case, however, both pyridine substituents are bonded to the same amine N atom (Heitzmann *et al.*, 2009 ▸). The C—C chain length between the N atoms in the di­amine backbone of these ligands allows for the formation of five-membered chelate rings. Hancock has shown the formation of five-membered chelate rings to be more favourable for larger metal ions than for smaller metal ions (Hancock & Martell, 1989 ▸). The ligand precursor, H_2_bpcd (C), for the title compound is similar to A and B, but it incorporates the ethyl­enedi­amine backbone into a cyclo­hexyl group. Restricted rotation about the C—C bonds in the cyclo­hexane ring fixes the positions of the trans di­amine nitro­gen atoms and favourably preorganizes these donor groups for metal ion complexation. Consequently, the trans amine groups are constrained into a conformation that is pre-oriented favorably for binding and results in a complex of increased stability (Rizkalla *et al.*, 1987 ▸; Choppin *et al.*, 2006 ▸; Ogden *et al.*, 2012 ▸). In contrast, H_2_bppd (D) features a 1,3-di­amino­propane backbone that provides greater flexibility compared to A, B, or C with their shorter backbones. Further, the increased chain length of the propyl­ene linker allows a six-membered chelate ring to form upon metal complexation. Formation of six-membered chelate rings in complexes with smaller metal ions has been shown to increase the stability of the complex relative to five-membered rings (Hancock & Martell, 1989 ▸). Here, we report the structure of a Co^III^ complex with bpcd^2−^, C.

## Structural commentary   

**Figure 2:**
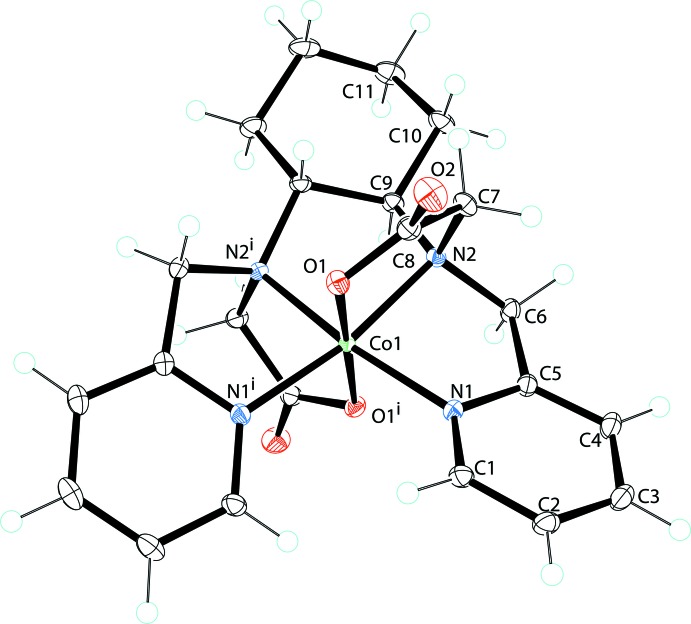
View of the cation of the title structure, [Co(bpcd)]^+^. Here and in subsequent figures, displacement ellipsoids are shown at the 50% probability level. H atoms are shown as circles of arbitrary size. [Symmetry code: (i) −*x* + 1, −*y* + 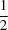, *z*.]

The structure of the [Co(bpcd)]^+^ cation in the title compound is shown in Fig. 2 ▸ and selected geometric parameters are listed in Table 1 ▸. The cation is very similar to the structures of the [Co(bped)]^+^ and [Co(bppd)]^+^ complex ions. Nearly all of the Co—O_ac_ bond lengths for the five structures given in Table 1 ▸ are within experimental error of each other. One of the Co—O_ac_ bond lengths in the [Co(bppd)]^+^ cation, however, is slightly shorter than the others. The C—O and C=O bond lengths are also quite similar. There are, however, some variations in the bond lengths and angles as shown in Tables 1 ▸ and 2 ▸. The Co—N_am_ bond length in the [Co(bpcd)]^+^ cation is slightly shorter than the Co—N_am_ bond lengths reported for the two [Co(bppd)]^+^ cations given in Table 1 ▸. They are, however, slightly longer than those reported for the [Co(bped)]^+^ structures. Similarly, the N_am1_—Co—N_am2_ bond angle in [Co(bpcd)]^+^ is close to ideal (90°), whereas the N_am1_—Co—N_am2_ angles in the [Co(bppd)]^+^ structures are somewhat larger than ideal and somewhat smaller than ideal in the [Co(bped)]^+^ structures (Table 2 ▸). The O_ac1_—Co—O_ac2_ bond angles for the five structures in Table 2 ▸ are all close to ideal (180°), with the largest deviation from linearity observed in the[Co(bpcd)]^+^ cation. The 176.1° O_ac1_—Co—O_ac2_ bond angle in [Co(bpcd)]^+^ is 2° smaller than the average (178.5°) of the bond angles reported for the [Co(bped)]^+^ and [Co(bppd)]^+^ cations. Finally, the Co^III^ in the title compound is situated directly in the N_4_ plane of the equatorial nitro­gen atoms, whereas in three of the other four structures the Co^III^ lays slightly out-of the plane (Table 1 ▸). The solid-state structural parameters for [Co(bpcd)]^+^, which are very similar to those for Co(bped)^+^, suggest that the ligand precusor H_2_(bpcd), with its preorganized arrangement, may provide greater metal ion complex stability as well as be selective for actinides(III) over lanthanides(III) as demonstrated for *gem*-H_2_(bped). (Heitzmann *et al.*, 2009 ▸)

## Supra­molecular features   

**Figure 3:**
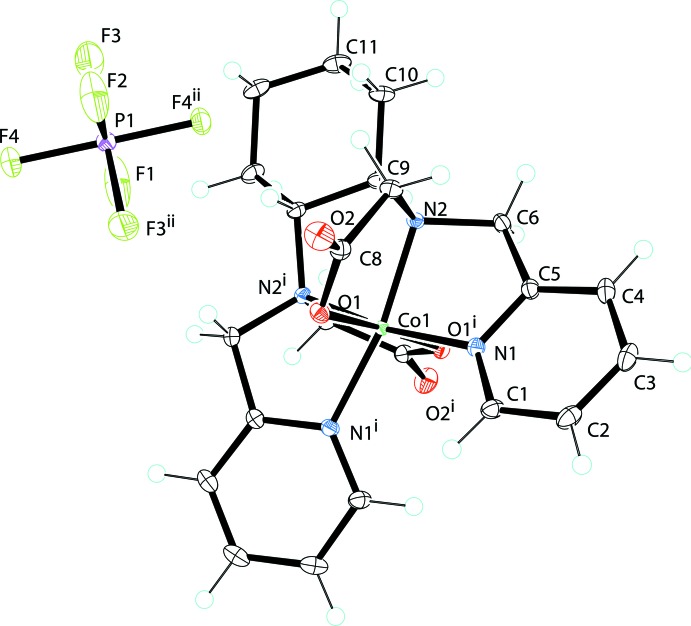
View of the molecular components of the title structure, [Co(bpcd)]PF_6_. [Symmetry code: (i) −*x* + 1, −*y* + 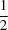, *z*.]

The structure of the title compound (Fig. 3 ▸) exists in the solid state as an intricate network of anions and cations closely associated through many short inter­actions. Hydrogen-bonding inter­actions are listed in Table 3 ▸. Each PF_6_^−^ anion is in close contact with six cations: three of the four unique F atoms inter­act with two neighboring cations while the remaining atom, F4, has a long inter­action (2.29 Å) with only the C—H9*A* bond of the cyclo­hexyl ring of one cation. This F4⋯H9*A* inter­action is the shortest of the F⋯H inter­actions present with two other weaker F⋯H inter­actions of 2.49 (F1⋯H10*A*) and 2.64 Å (F1⋯H9*A*) to cyclo­hexyl H atoms. There are also several inter­actions between pyrdidyl ring H atoms and carboxyl­ate O atoms from neighboring cations, *i.e.* a 2.408 Å inter­action with Co-bound oxygen O1, and a 2.700 Å inter­action with terminal oxygen O2. The short inter­action has a C—H⋯O angle of 140.7° so it does not appear in Table 3 ▸. There also exists π–π stacking for each of the two pyridyl rings with neighboring cations stacked anti­parallel. Each has a distance of 3.829 (13) Å between ring centroids.

## Database survey   

There is very little information in the literature about H_2_bpcd and its metal complexes. There is a structurally characterized hepta­coordinate [Fe^II^(H_2_bpcd)(C_3_H_6_O)](ClO_4_)_2_ complex with trans pyridine N atoms and cis carb­oxy­lic acid groups (Oddon *et al.*, 2012 ▸). In that case, Fe^II^ is coordinated in a distorted penta­gonal–bipyramidal geometry with an unusual N_4_O_3_ donor atom set, including a bound acetone mol­ecule. The carb­oxy­lic acid moieties are fully protonated with the H_2_bpcd ligand coordinating through the carbonyl O atoms, which reside in the equatorial plane. The coordinating amine N atoms also lie in this plane, whereas the pyridyl N atoms are coordinating at the axial positions. This unique arrangement results in longer Fe—O and Fe—N_py_ bonds than are typically observed. In the present case, a fully deprotonated bpcd^2−^ ligand binds Co^III^ in a pseudo-octa­hedral fashion with trans acetate groups to form a hexa­coordinate complex.

Although only one structure of a metal–H_2_bpcd complex has been reported in the literature, there are several structures reported for related pseudo-octa­hedral Co^III^ complexes with bis-2-pyridyl­methyl substituted di­amino di­acetic acids, *i.e.* H_2_bped (A) and H_2_bppd (D) in Fig. 1 ▸. We previously reported the structure of [Co(bppd)]PF_6_ (McLauchlan *et al.*, 2013 ▸), and there are two structural reports for the [Co(bped)]^+^ complex ion with different counter-ions, *e.g.* BF_4_^−^ and PF_6_^−^ (Mandel & Douglas, 1989 ▸; Caravan *et al.*, 1997*a* ▸). In these cases, the Co^III^–bppd^2−^ and Co^III^–bped^2−^ complexes form similar hexa­dentate structures with acetate O atoms in a trans orientation and pyridyl N atoms in a cis orientation.

## Synthesis and crystallization   

H_2_bpcd (C) was prepared from *trans*-1,2-di­amino­cyclo­hexane using the procedure reported for H_2_bppd (D) (Kissel *et al.*, 2014 ▸). The title compound was prepared using methods analogous to those previously reported for [Co(bppd)]PF_6_ (McLauchlan *et al.*, 2013 ▸). Crystals suitable for diffraction were isolated by slow evaporation of a saturated aceto­nitrile solution (yield: 120 mg, 0.20 mmol, 40%).

Analysis observed (calculated) for CoC_22_H_28_N_4_O_4_PF_6_: C 42.56 (43.00), H 3.85 (4.26), N 8.94 (9.11). IR (ν cm^−1^, KBr): 3048 (*m*, C—H aryl str), 2945 (*m*, CH_2_ str), 1665 (*vs*, COO^−^ str), 1612 (*m*, py str), 1477 (*w*, py str), 1445 (*m*, CH_2_ def), 1384 (*s*, COO^−^ str).

## Refinement   

Crystal data, data collection and structure refinement details are summarized in Table 4 ▸. The structure of the title complex can be solved and refined in *Ibca* with well-separated cations and anions. There is a small amount of disorder that can be modelled for the PF_6_^−^ anion. F2 and F3 can be moved in the plane. *R*1 can be reduced to 0.0252 by modeling this disorder, but the occupancy is less than 10% and results in a less chemically satisfactory PF_6_^−^ anion. Therefore, the disorder was not modelled. All H atoms were placed geometrically (C—H = 0.93–0.97 Å) and refined using a riding model.

## Supplementary Material

Crystal structure: contains datablock(s) I. DOI: 10.1107/S2056989015005149/zl2616sup1.cif

Structure factors: contains datablock(s) I. DOI: 10.1107/S2056989015005149/zl2616Isup2.hkl

Supporting information file. DOI: 10.1107/S2056989015005149/zl2616Isup3.cdx

CCDC reference: 1053810

Additional supporting information:  crystallographic information; 3D view; checkCIF report

## Figures and Tables

**Table 1:** Bond distances () and experimental data for different [Co(bp*a*d)]^+^ structures

Bond ()	Co(bped)^+ a^	Co(bped)^+ b^	Co(bppd)^+ c^ 1	Co(bppd)^+ c^ 2	Co(bpcd)^+ d^
CoO_*ac*1_	1.888(1)	1.878(2)	1.8828(11)	1.8875(10)	1.8869(8)
CoO_*ac*2_	1.889(2)	1.888(2)	1.8899(11)	1.8830(11)	*
CoN_*am*1_	1.941(2)	1.937(2)	1.9625(13)	1.9654(12)	1.9548(9)
CoN_*am*2_	1.974(2)	1.941(2)	1.9641(13)	1.9645(12)	*
CoN_pyr1_	1.944(2)	1.960(2)	1.9484(13)	1.9403(13)	1.9448(9)
CoN_pyr2_	1.954(2)	1.958(2)	1.9397(13)	1.9576(13)	*
CO_*ac*1_	1.294(2)	1.298(4)	1.2973(18)	1.3054(18)	1.3029(13)
CO_*ac*1_	1.212(3)	1.218(3)	1.2265(18)	1.219(2)	1.2212(14)
CO_*ac*2_	1.289(3)	1.299(3)	1.3035(19)	1.2971(19)	*
CO_*ac*2_	1.210(3)	1.213(3)	1.2201(19)	0.0030(6)	*
Co above N/N/N/N plane	0.000	0.012	0.0026(6)	0.0030(6)	0**
Temp, K	298	293	100	100	100

Notes: (*a*) Mandel Douglas (1989 ▸); (*b*) Caravan *et al.* (1997*a* ▸); (*c*) two cations in asymmetric unit (McLauchlan *et al.*, 2013 ▸); (*d*) this work; (*) N/A symmetry equivalent; () standard uncertainty unavailable; (**) N/A sits on a special position.

**Table 2:** Selected bond angles () for different [Co(bp*a*d)]^+^ structures

Angle,	Co(bped)^+ a^	Co(bped)^+ b^	Co(bppd)^+ c^ 1	Co(bppd)^+ c^ 2	Co(bpcd)^+ d^
O_*ac*1_CoO_*ac*2_	178.8(1)	178.53(8)	178.47(5)	178.36(5)	176.08(5)
N_*am*1_CoN_*am*2_	82.0(1)	88.87(9)	95.91(5)	95.92(5)	89.33(5)
N_pyr1_CoN_pyr2_	82.3(1)	107.01(9)	98.52(6)	98.55(5)	106.74(5)
N_*am*1_CoN_pyr1_	89.3(1)	82.14(9)	82.36(6)	83.23(5)	82.17(4)
N_*am*2_CoN_pyr2_	107.0(1)	82.51(9)	83.28(6)	82.39(5)	*
N_*am*1_CoO_*ac*1_	86.9(1)	87.36(9)	88.81(5)	87.96(5)	87.84(4)
N_pyr1_CoO_*ac*1_	92.8(1)	92.34(8)	86.51(5)	87.72(5)	89.92(4)
OCO_*ac*_	124.4(2)	123.9(3)	123.87(14)	123.80(14)	124.95(10)
	124.7(2)	124.8(3)	123.95(15)	123.82(14)	*
C(O)O_*ac*_Co	116.4(1)	116.4(2)	114.32(9)	115.33(10)	114.57(7)
	115.9(1)	115.3(2)	115.11(10)	114.38(9)	*

Notes: (*a*) Mandel Douglas (1989 ▸); (*b*) Caravan *et al.* (1997*a* ▸); (*c*) two cations in asymmetric unit (McLauchlan *et al.*, 2013 ▸); (*d*) this work; (*) N/A symmetry equivalent; (**) N/A sits on a special position.

**Table 3:** Hydrogen-bond geometry (, )

*D*H*A*	*D*H	H*A*	*D* *A*	*D*H*A*
C1H1*A*O2^ii^	0.95	2.84	3.4475(15)	122
C2H2*A*F3^iii^	0.95	2.51	3.2928(15)	139
C4H4*A*O2^iv^	0.95	2.70	3.5907(15)	157
C6H6*A*F2^v^	0.99	2.52	3.4243(13)	152
C6H6*B*F1^vi^	0.99	2.74	3.3824(13)	123
C6H6*B*F3^vi^	0.99	2.84	3.8229(18)	170
C7H7*A*F4^vii^	0.99	2.68	3.3879(13)	128
C7H7*A*F4^iv^	0.99	2.67	3.2436(13)	117
C7H7*B*F3^v^	0.99	2.62	3.4982(16)	147
C9H9*A*F1^vi^	1.00	2.64	3.2790(12)	122
C9H9*A*F4^vi^	1.00	2.29	3.2336(13)	157
C10H10*A*F1^vi^	0.99	2.49	3.1429(15)	123
C10H10*A*F2^v^	0.99	2.35	3.0728(14)	129
C10H10*B*F4^iv^	0.99	2.77	3.5399(14)	135

Symmetry codes: (ii) 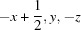; (iii) 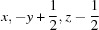; (iv) 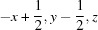; (v) 
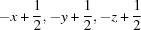
; (vi) 
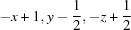
; (vii) 
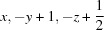
.

**Table 4:** Experimental details

Crystal data	
Chemical formula	[Co(C_22_H_26_N_4_O_4_)]PF_6_
*M*_r_	614.37
Crystal system, space group	Orthorhombic, *I* *b* *c* *a*
Temperature (K)	100
*a*, *b*, *c* ()	13.9848(4), 14.6221(4), 22.2177(6)
*V* (^3^)	4543.2(2)
*Z*	8
Radiation type	Mo *K*
(mm^1^)	0.92
Crystal size (mm)	0.44 0.36 0.21

Data collection	
Diffractometer	Bruker APEXII equipped with a CCD detector
Absorption correction	Multi-scan (*SADABS*; Bruker, 2008 ▸)
*T*_min_, *T*_max_	0.691, 0.834
No. of measured, independent and observed [*I* > 2(*I*)] reflections	57644, 3630, 3401
*R*_int_	0.017
(sin /)_max_ (^1^)	0.725

Refinement	
*R*[*F* ^2^ > 2(*F* ^2^)], *wR*(*F* ^2^), *S*	0.027, 0.079, 1.12
No. of reflections	3630
No. of parameters	174
H-atom treatment	H-atom parameters constrained
_max_, _min_ (e ^3^)	0.66, 0.52

Computer programs: *APEX2* and *SAINT* (Bruker, 2008 ▸), *SHELXS97* (Sheldrick, 2008 ▸), *SHELXL2014* (Sheldrick, 2015 ▸), *ORTEP-3 for Windows* (Farrugia, 2012 ▸), *enCIFer* (Allen *et al.*, 2004 ▸) and *publCIF* (Westrip, 2010 ▸).

## supplementary crystallographic information

### Crystal data

**Table:**

[Co(C_22_H_26_N_4_O_4_)]PF_6_	*D*_x_ = 1.796 Mg m^−^^3^
*M**_r_* = 614.37	Mo *K*α radiation, λ = 0.71073 Å
Orthorhombic, *I**b**c**a*	Cell parameters from 9742 reflections
*a* = 13.9848 (4) Å	θ = 2.7–31.0°
*b* = 14.6221 (4) Å	µ = 0.92 mm^−^^1^
*c* = 22.2177 (6) Å	*T* = 100 K
*V* = 4543.2 (2) Å^3^	Parallelipiped, translucent dark red
*Z* = 8	0.44 × 0.36 × 0.21 mm
*F*(000) = 2512	

### Data collection

**Table:**

Bruker APEXII diffractometer equipped with a CCD detector	3630 independent reflections
Radiation source: fine-focus sealed tube	3401 reflections with *I* > 2σ(*I*)
Graphite monochromator	*R*_int_ = 0.017
Detector resolution: 8.3333 pixels mm^-1^	θ_max_ = 31.0°, θ_min_ = 1.8°
φ and ω scans	*h* = −20→20
Absorption correction: multi-scan (*SADABS*; Bruker, 2008)	*k* = −21→21
*T*_min_ = 0.691, *T*_max_ = 0.834	*l* = −32→32
57644 measured reflections	

### Refinement

**Table:**

Refinement on *F*^2^	Primary atom site location: structure-invariant direct methods
Least-squares matrix: full	Secondary atom site location: difference Fourier map
*R*[*F*^2^ > 2σ(*F*^2^)] = 0.027	Hydrogen site location: inferred from neighbouring sites
*wR*(*F*^2^) = 0.079	H-atom parameters constrained
*S* = 1.12	*w* = 1/[σ^2^(*F*_o_^2^) + (0.0415*P*)^2^ + 5.6191*P*] where *P* = (*F*_o_^2^ + 2*F*_c_^2^)/3
3630 reflections	(Δ/σ)_max_ = 0.001
174 parameters	Δρ_max_ = 0.66 e Å^−^^3^
0 restraints	Δρ_min_ = −0.52 e Å^−^^3^

### Special details

**Table:**

Geometry. All e.s.d.'s (except the e.s.d. in the dihedral angle between two l.s. planes) are estimated using the full covariance matrix. The cell e.s.d.'s are taken into account individually in the estimation of e.s.d.'s in distances, angles and torsion angles; correlations between e.s.d.'s in cell parameters are only used when they are defined by crystal symmetry. An approximate (isotropic) treatment of cell e.s.d.'s is used for estimating e.s.d.'s involving l.s. planes.
Refinement. Refinement of *F*^2^ against ALL reflections. The weighted *R*-factor *wR* and goodness of fit *S* are based on *F*^2^, conventional *R*-factors *R* are based on *F*, with *F* set to zero for negative *F*^2^. The threshold expression of *F*^2^ > σ(*F*^2^) is used only for calculating *R*-factors(gt) *etc*. and is not relevant to the choice of reflections for refinement. *R*-factors based on *F*^2^ are statistically about twice as large as those based on *F*, and *R*- factors based on ALL data will be even larger. There is a small amount of disorder that can be modeled for the PF_6_ anion. F2 and F3 can be moved in the plane, as one might imagine. R1 can be reduced to 0.0252 by modeling it, but the occupancy is less than 10% and results in a less chemically satisfactory PF_6_ anion. Therefore, the disorder was not modeled.

### Fractional atomic coordinates and isotropic or equivalent isotropic displacement parameters (Å^2^)

**Table:**

	*x*	*y*	*z*	*U*_iso_*/*U*_eq_	
Co1	0.5000	0.2500	0.09254 (2)	0.00761 (6)	
N1	0.43958 (7)	0.15960 (6)	0.04005 (4)	0.01066 (16)	
N2	0.43848 (6)	0.17734 (6)	0.15480 (4)	0.00930 (16)	
O1	0.38813 (6)	0.32200 (5)	0.08964 (3)	0.01162 (15)	
O2	0.23055 (6)	0.30823 (7)	0.10495 (4)	0.01942 (18)	
C1	0.41181 (8)	0.16885 (8)	−0.01758 (5)	0.01342 (19)	
H1A	0.4189	0.2265	−0.0369	0.016*	
C2	0.37301 (8)	0.09611 (8)	−0.04959 (5)	0.0159 (2)	
H2A	0.3555	0.1035	−0.0906	0.019*	
C3	0.36018 (8)	0.01258 (8)	−0.02102 (6)	0.0160 (2)	
H3A	0.3356	−0.0384	−0.0426	0.019*	
C4	0.38383 (8)	0.00449 (8)	0.03974 (5)	0.0145 (2)	
H4A	0.3729	−0.0511	0.0607	0.017*	
C5	0.42361 (7)	0.07927 (7)	0.06891 (5)	0.01135 (18)	
C6	0.44904 (8)	0.08027 (7)	0.13472 (5)	0.01223 (18)	
H6A	0.4055	0.0399	0.1577	0.015*	
H6B	0.5156	0.0590	0.1408	0.015*	
C7	0.33453 (7)	0.20455 (8)	0.15563 (5)	0.01188 (19)	
H7A	0.3164	0.2220	0.1971	0.014*	
H7B	0.2951	0.1513	0.1439	0.014*	
C8	0.31295 (8)	0.28378 (8)	0.11346 (5)	0.01202 (18)	
C9	0.49132 (7)	0.19846 (8)	0.21257 (5)	0.01084 (18)	
H9A	0.5551	0.1678	0.2102	0.013*	
C10	0.44229 (8)	0.16490 (8)	0.27004 (5)	0.0154 (2)	
H10A	0.4399	0.0972	0.2700	0.019*	
H10B	0.3758	0.1882	0.2714	0.019*	
C11	0.49688 (8)	0.19815 (10)	0.32563 (5)	0.0182 (2)	
H11A	0.4637	0.1772	0.3625	0.022*	
H11B	0.5621	0.1718	0.3256	0.022*	
P1	0.30339 (3)	0.5000	0.2500	0.01160 (8)	
F1	0.41712 (9)	0.5000	0.2500	0.0503 (5)	
F2	0.19013 (9)	0.5000	0.2500	0.0417 (4)	
F3	0.30336 (11)	0.49194 (7)	0.32124 (4)	0.0470 (3)	
F4	0.30308 (5)	0.60985 (5)	0.25526 (4)	0.01831 (15)	

### Atomic displacement parameters (Å^2^)

**Table:**

	*U*^11^	*U*^22^	*U*^33^	*U*^12^	*U*^13^	*U*^23^
Co1	0.00914 (10)	0.00719 (10)	0.00650 (10)	−0.00083 (6)	0.000	0.000
N1	0.0118 (4)	0.0103 (4)	0.0099 (4)	−0.0015 (3)	−0.0002 (3)	−0.0010 (3)
N2	0.0103 (4)	0.0095 (4)	0.0081 (4)	−0.0005 (3)	0.0000 (3)	0.0010 (3)
O1	0.0113 (3)	0.0106 (3)	0.0130 (3)	0.0008 (3)	−0.0004 (3)	0.0018 (3)
O2	0.0119 (4)	0.0223 (4)	0.0241 (4)	0.0033 (3)	−0.0017 (3)	0.0040 (3)
C1	0.0140 (4)	0.0167 (5)	0.0095 (4)	−0.0025 (4)	0.0000 (3)	−0.0006 (3)
C2	0.0133 (5)	0.0222 (5)	0.0123 (4)	−0.0031 (4)	0.0001 (4)	−0.0053 (4)
C3	0.0117 (4)	0.0167 (5)	0.0198 (5)	−0.0014 (4)	0.0007 (4)	−0.0087 (4)
C4	0.0129 (4)	0.0105 (4)	0.0200 (5)	−0.0008 (3)	0.0003 (4)	−0.0036 (4)
C5	0.0111 (4)	0.0100 (4)	0.0129 (4)	−0.0007 (3)	0.0004 (3)	−0.0008 (3)
C6	0.0156 (4)	0.0087 (4)	0.0124 (4)	−0.0011 (3)	−0.0010 (4)	0.0014 (3)
C7	0.0095 (4)	0.0143 (5)	0.0118 (4)	−0.0003 (3)	0.0000 (3)	0.0026 (3)
C8	0.0125 (4)	0.0125 (4)	0.0111 (4)	0.0001 (4)	−0.0010 (3)	−0.0002 (3)
C9	0.0115 (4)	0.0133 (5)	0.0077 (4)	−0.0004 (3)	−0.0007 (3)	0.0013 (3)
C10	0.0163 (5)	0.0208 (5)	0.0092 (4)	−0.0020 (4)	0.0011 (4)	0.0037 (4)
C11	0.0182 (5)	0.0277 (6)	0.0087 (4)	0.0011 (4)	−0.0006 (4)	0.0031 (4)
P1	0.01124 (17)	0.01151 (17)	0.01203 (17)	0.000	0.000	0.00031 (13)
F1	0.0127 (5)	0.0204 (6)	0.1178 (16)	0.000	0.000	−0.0134 (8)
F2	0.0128 (5)	0.0206 (6)	0.0916 (13)	0.000	0.000	−0.0056 (7)
F3	0.1008 (10)	0.0246 (5)	0.0157 (4)	0.0046 (5)	−0.0073 (5)	−0.0001 (3)
F4	0.0176 (3)	0.0116 (3)	0.0258 (4)	0.0001 (2)	−0.0029 (3)	−0.0010 (3)

### Geometric parameters (Å, º)

**Table:**

Co1—O1^i^	1.8869 (8)	C5—C6	1.5050 (15)
Co1—O1	1.8869 (8)	C6—H6A	0.9900
Co1—N1	1.9548 (9)	C6—H6B	0.9900
Co1—N1^i^	1.9548 (9)	C7—C8	1.5201 (15)
Co1—N2	1.9448 (9)	C7—H7A	0.9900
Co1—N2^i^	1.9449 (9)	C7—H7B	0.9900
N1—C1	1.3448 (14)	C9—C9^i^	1.527 (2)
N1—C5	1.3567 (14)	C9—C10	1.5300 (15)
N2—C6	1.4951 (14)	C9—H9A	1.0000
N2—C7	1.5073 (14)	C10—C11	1.5312 (16)
N2—C9	1.5130 (13)	C10—H10A	0.9900
O1—C8	1.3029 (13)	C10—H10B	0.9900
O2—C8	1.2212 (14)	C11—C11^i^	1.519 (3)
C1—C2	1.3898 (15)	C11—H11A	0.9900
C1—H1A	0.9500	C11—H11B	0.9900
C2—C3	1.3882 (17)	P1—F2	1.5840 (13)
C2—H2A	0.9500	P1—F3	1.5872 (9)
C3—C4	1.3949 (17)	P1—F3^ii^	1.5873 (9)
C3—H3A	0.9500	P1—F1	1.5905 (14)
C4—C5	1.3873 (15)	P1—F4	1.6106 (7)
C4—H4A	0.9500	P1—F4^ii^	1.6106 (7)
			
O1^i^—Co1—O1	176.08 (5)	C5—C6—H6B	110.5
O1^i^—Co1—N2	94.95 (4)	H6A—C6—H6B	108.7
O1—Co1—N2	87.84 (4)	N2—C7—C8	112.65 (8)
O1^i^—Co1—N2^i^	87.84 (4)	N2—C7—H7A	109.1
O1—Co1—N2^i^	94.95 (4)	C8—C7—H7A	109.1
N2—Co1—N2^i^	89.33 (5)	N2—C7—H7B	109.1
O1^i^—Co1—N1	87.75 (4)	C8—C7—H7B	109.1
O1—Co1—N1	89.92 (4)	H7A—C7—H7B	107.8
N2—Co1—N1	82.17 (4)	O2—C8—O1	124.95 (10)
N2^i^—Co1—N1	170.04 (4)	O2—C8—C7	120.39 (10)
O1^i^—Co1—N1^i^	89.92 (4)	O1—C8—C7	114.65 (9)
O1—Co1—N1^i^	87.75 (4)	N2—C9—C9^i^	106.21 (7)
N2—Co1—N1^i^	170.04 (4)	N2—C9—C10	115.07 (9)
N2^i^—Co1—N1^i^	82.17 (4)	C9^i^—C9—C10	112.82 (7)
N1—Co1—N1^i^	106.74 (5)	N2—C9—H9A	107.5
C1—N1—C5	119.30 (9)	C9^i^—C9—H9A	107.5
C1—N1—Co1	128.67 (8)	C10—C9—H9A	107.5
C5—N1—Co1	112.01 (7)	C9—C10—C11	110.36 (9)
C6—N2—C7	110.46 (8)	C9—C10—H10A	109.6
C6—N2—C9	113.49 (8)	C11—C10—H10A	109.6
C7—N2—C9	114.01 (8)	C9—C10—H10B	109.6
C6—N2—Co1	105.23 (6)	C11—C10—H10B	109.6
C7—N2—Co1	106.93 (6)	H10A—C10—H10B	108.1
C9—N2—Co1	106.01 (6)	C11^i^—C11—C10	110.22 (9)
C8—O1—Co1	114.57 (7)	C11^i^—C11—H11A	109.6
N1—C1—C2	121.54 (10)	C10—C11—H11A	109.6
N1—C1—H1A	119.2	C11^i^—C11—H11B	109.6
C2—C1—H1A	119.2	C10—C11—H11B	109.6
C3—C2—C1	119.32 (10)	H11A—C11—H11B	108.1
C3—C2—H2A	120.3	F2—P1—F3	89.98 (6)
C1—C2—H2A	120.3	F2—P1—F3^ii^	89.98 (6)
C2—C3—C4	119.11 (10)	F3—P1—F3^ii^	179.97 (11)
C2—C3—H3A	120.4	F2—P1—F1	180.0
C4—C3—H3A	120.4	F3—P1—F1	90.02 (6)
C5—C4—C3	118.70 (11)	F3^ii^—P1—F1	90.02 (6)
C5—C4—H4A	120.6	F2—P1—F4	89.84 (3)
C3—C4—H4A	120.6	F3—P1—F4	90.09 (5)
N1—C5—C4	121.85 (10)	F3^ii^—P1—F4	89.91 (5)
N1—C5—C6	114.31 (9)	F1—P1—F4	90.16 (3)
C4—C5—C6	123.80 (10)	F2—P1—F4^ii^	89.84 (3)
N2—C6—C5	106.01 (8)	F3—P1—F4^ii^	89.91 (5)
N2—C6—H6A	110.5	F3^ii^—P1—F4^ii^	90.09 (5)
C5—C6—H6A	110.5	F1—P1—F4^ii^	90.16 (3)
N2—C6—H6B	110.5	F4—P1—F4^ii^	179.69 (6)
			
N2—Co1—O1—C8	−17.97 (8)	N1—C5—C6—N2	27.33 (12)
N2^i^—Co1—O1—C8	−107.11 (8)	C4—C5—C6—N2	−150.39 (10)
N1—Co1—O1—C8	64.20 (8)	C6—N2—C7—C8	−119.41 (9)
N1^i^—Co1—O1—C8	170.96 (8)	C9—N2—C7—C8	111.40 (10)
C5—N1—C1—C2	−4.46 (16)	Co1—N2—C7—C8	−5.42 (10)
Co1—N1—C1—C2	177.37 (8)	Co1—O1—C8—O2	−162.99 (10)
N1—C1—C2—C3	1.77 (17)	Co1—O1—C8—C7	18.41 (12)
C1—C2—C3—C4	1.97 (17)	N2—C7—C8—O2	173.28 (10)
C2—C3—C4—C5	−2.94 (16)	N2—C7—C8—O1	−8.05 (13)
C1—N1—C5—C4	3.43 (16)	C6—N2—C9—C9^i^	156.93 (9)
Co1—N1—C5—C4	−178.11 (8)	C7—N2—C9—C9^i^	−75.42 (11)
C1—N1—C5—C6	−174.34 (9)	Co1—N2—C9—C9^i^	41.93 (10)
Co1—N1—C5—C6	4.12 (11)	C6—N2—C9—C10	−77.49 (11)
C3—C4—C5—N1	0.28 (16)	C7—N2—C9—C10	50.16 (12)
C3—C4—C5—C6	177.83 (10)	Co1—N2—C9—C10	167.51 (8)
C7—N2—C6—C5	69.87 (10)	N2—C9—C10—C11	−174.45 (9)
C9—N2—C6—C5	−160.66 (8)	C9^i^—C9—C10—C11	−52.36 (14)
Co1—N2—C6—C5	−45.20 (9)	C9—C10—C11—C11^i^	58.00 (14)

Symmetry codes: (i) −*x*+1, −*y*+1/2, *z*; (ii) *x*, −*y*+1, −*z*+1/2.

### Hydrogen-bond geometry (Å, º)

**Table:**

*D*—H···*A*	*D*—H	H···*A*	*D*···*A*	*D*—H···*A*
C1—H1*A*···O2^iii^	0.95	2.84	3.4475 (15)	122
C2—H2*A*···F3^iv^	0.95	2.51	3.2928 (15)	139
C4—H4*A*···O2^v^	0.95	2.70	3.5907 (15)	157
C6—H6*A*···F2^vi^	0.99	2.52	3.4243 (13)	152
C6—H6*B*···F1^vii^	0.99	2.74	3.3824 (13)	123
C6—H6*B*···F3^vii^	0.99	2.84	3.8229 (18)	170
C7—H7*A*···F4^ii^	0.99	2.68	3.3879 (13)	128
C7—H7*A*···F4^v^	0.99	2.67	3.2436 (13)	117
C7—H7*B*···F3^vi^	0.99	2.62	3.4982 (16)	147
C9—H9*A*···F1^vii^	1.00	2.64	3.2790 (12)	122
C9—H9*A*···F4^vii^	1.00	2.29	3.2336 (13)	157
C10—H10*A*···F1^vii^	0.99	2.49	3.1429 (15)	123
C10—H10*A*···F2^vi^	0.99	2.35	3.0728 (14)	129
C10—H10*B*···F4^v^	0.99	2.77	3.5399 (14)	135

Symmetry codes: (ii) *x*, −*y*+1, −*z*+1/2; (iii) −*x*+1/2, *y*, −*z*; (iv) *x*, −*y*+1/2, *z*−1/2; (v) −*x*+1/2, *y*−1/2, *z*; (vi) −*x*+1/2, −*y*+1/2, −*z*+1/2; (vii) −*x*+1, *y*−1/2, −*z*+1/2.
